# A structured hands-on CSF diagnostic training module improves diagnostic knowledge in medical students: a prospective pre–post study

**DOI:** 10.1186/s12909-026-09990-2

**Published:** 2026-07-22

**Authors:** Yang Liu, Steffen Kottackal, Wenlin Hao

**Affiliations:** 1 Department of Neurology, Saarland University, Kirrberger Straße, Homburg, Saar, 66421 Germany; 2 SHG Klinikum Saarbrücken Psychiatrie, Saarbrücken, Germany

**Keywords:** Medical education, Neurology, Cerebrospinal fluid, Practical training, Pre–post study, Diagnostic skills

## Abstract

**Background:**

Cerebrospinal fluid (CSF) diagnostics is an essential component of neurological practice and requires not only theoretical knowledge but also the ability to interpret cytological findings and integrate them into clinical reasoning. Structured, practice-oriented approaches for teaching CSF diagnostics in undergraduate medical education remain limited. This study evaluated the impact of a structured, hands-on training module in CSF diagnostics on medical students’ knowledge acquisition and application of diagnostic knowledge.

**Methods:**

This prospective educational intervention study included fourth-year medical students participating in a mandatory neurology practical course. The intervention consisted of a pre-test, a structured theoretical session, a practical microscopy session using authentic CSF samples, and a post-test conducted during a single teaching session. Knowledge acquisition was assessed using pseudonymized pre- and post-tests covering CSF-related knowledge domains including indications for lumbar puncture, cytological findings, diagnostic interpretation, microscopy-based diagnosis and procedural aspects of CSF diagnostics. Student evaluations were collected immediately after the session using an anonymous questionnaire. Paired data were analyzed using the Wilcoxon signed-rank test and McNemar’s test, as appropriate.

**Results:**

Eighty-four medical students completed both assessments. Median test scores increased from 3 to 6 correct answers following the intervention (Wilcoxon signed-rank test: Z = − 7.886, *p* < 0.001). Improvements were observed particularly in procedural knowledge, diagnostic interpretation, cytological assessment, and microscopy-based diagnosis. Baseline performance regarding indications for lumbar puncture was already high and showed no significant change. Student evaluations indicated positive perceptions of the integration of theoretical and practical components.

**Conclusions:**

A structured, hands-on CSF diagnostics training module was associated with improved short-term knowledge acquisition and application of diagnostic knowledge related to CSF diagnostics among undergraduate medical students. Practice-oriented teaching approaches may complement traditional neurology education and support clinically relevant learning.

**Supplementary Information:**

The online version contains supplementary material available at 10.1186/s12909-026-09990-2.

## Introduction

Cerebrospinal fluid (CSF) analysis is a cornerstone of neurological diagnostics and contributes substantially to the diagnosis and differential diagnosis of infectious, inflammatory, vascular, autoimmune, and neuroimmunological disorders of the central nervous system. Effective use of CSF diagnostics requires considerably more than knowledge of laboratory parameters. Clinicians must recognize cytological abnormalities, interpret laboratory findings in conjunction with the patient’s clinical presentation, neuroimaging, and other laboratory investigations, and integrate this information into diagnostic decision-making. Because laboratory specialists often have limited access to the complete clinical context, whereas treating physicians may rely heavily on laboratory reports, accurate diagnosis ultimately depends on the clinician’s ability to critically interpret CSF findings rather than simply apply laboratory conclusions.

Recognizing the growing burden of neurological disease, contemporary undergraduate neurology education has increasingly shifted from knowledge transmission toward competency-based training. The American Academy of Neurology (AAN) core curriculum emphasizes that medical students should acquire not only procedural skills, such as performing lumbar puncture, but also analytical skills, including interpretation of diagnostic investigations, formulation of differential diagnoses, clinical reasoning, and avoidance of diagnostic errors [1]. These competencies are considered fundamental learning objectives for all graduating medical students, regardless of their future specialty.

Consistent with this educational paradigm, active and experiential learning approaches have been shown to improve learner engagement, knowledge acquisition, clinical reasoning, and long-term retention compared with traditional lecture-based instruction [2, 3]. Experiential learning theory further proposes that knowledge is most effectively acquired when learners actively engage with authentic clinical tasks and reflect on their experiences. A recent scoping review of undergraduate neurology education, which synthesized evidence from 102 educational studies, demonstrated that experiential teaching has become a major instructional approach in neurology education. However, the review also highlighted substantial heterogeneity in educational interventions, limited evidence-based guidance for curriculum development, and a predominance of educational outcomes focusing on student perception and factual knowledge rather than clinical competence [4].

Although practical skills such as lumbar puncture are increasingly incorporated into neurology curricula [5–7], comparatively less attention has been directed toward teaching the interpretation of authentic CSF findings within a clinically relevant diagnostic framework. Existing educational approaches frequently emphasize procedural aspects of CSF acquisition or theoretical knowledge of laboratory parameters, whereas opportunities for students to independently examine real CSF cytology preparations, recognize pathological cellular patterns, integrate microscopic observations with clinical information, and formulate diagnostic conclusions are rarely described. Consequently, students may acquire factual knowledge of CSF analysis without developing the diagnostic reasoning required for real-world neurological practice.

To address this educational need, we developed a structured CSF laboratory practical course integrating focused theoretical instruction with hands-on microscopy using authentic patient CSF specimens. During the practical session, students individually examined CSF cytology preparations, identified pathological findings, interpreted their observations, and discussed their diagnostic implications in a supervised group setting. The intervention was designed according to competency-based educational principles to promote active participation, strengthen diagnostic reasoning, and bridge the gap between theoretical knowledge and clinical diagnostic reasoning.

The aim of this study was to evaluate whether a structured, hands-on CSF diagnostics training module improves short-term knowledge acquisition and application of diagnostic knowledge related to CSF diagnostics in undergraduate medical students.

## Methods

### Study design and educational setting

This prospective educational intervention study evaluated a competency-based CSF diagnostics training module conducted at the Department of Neurology, Saarland University. All fourth-year medical students during the summer semester of 2023 were eligible for inclusion in the study. During the semester, students participated in a structured neurology teaching program consisting of weekly lectures (3 h/week over 14 weeks) delivered concurrently with bedside practical teaching sessions (3 h/week over 14 weeks). The CSF diagnostics module represented one component of the practical neurology curriculum and was integrated into the ongoing teaching program.

A total of 84 students attended the course and completed both educational assessments. Teaching was delivered in person in groups of 6 students and facilitated by one of two instructors. Fourteen sessions were conducted over one semester, with one group participating per week.

Participation in the teaching session was mandatory because it formed part of the undergraduate neurology curriculum. However, participation in the educational evaluation (pre- and post-tests) and the use of assessment data for research purposes were voluntary. The quizzes were designed solely for educational evaluation, were not graded, did not contribute to course assessment, and no financial or academic incentives were provided for participation.

All procedures performed in this study involving human participants and human materials were in accordance with the ethical standards of the institutional research committee and with the “World Medical Association Declaration of Helsinki” (Paragraph 32) [8]. The study was approved by the local ethics committee (Ethics Committee of the Saarland Medical Association, identification number 08/11).

### Educational intervention

The intervention was designed according to competency-based educational principles. Rather than focusing primarily on factual knowledge acquisition, the learning objectives emphasized competencies required for real-world CSF diagnostics, including recognition of pathological cytological findings, interpretation of laboratory and microscopic observations, integration of these findings with clinical information, and formulation of likely diagnostic conclusions. During the practical session, students independently performed the analytical steps of the diagnostic process, including microscopic examination of authentic CSF specimens, recognition of pathological findings, interpretation of cytological observations, formulation of a likely diagnosis, and discussion of their diagnostic reasoning with the instructors and peers. Thus, the intervention targeted the development of analytical and diagnostic competencies rather than knowledge recall alone.

Teaching was delivered by two instructors, both neurologists with responsibility for routine clinical CSF diagnostics and experience in CSF cytological interpretation at the institution. The instructors jointly developed and delivered the educational materials. To ensure consistency across teaching sessions, standardized teaching materials and identical microscopy preparations were used throughout the course.

Each teaching session consisted of four consecutive components conducted during a single in-person session:

1. Pre-test (20 min): Students completed a paper-based pseudonymized multiple-choice test designed to assess baseline knowledge across seven domains: 1) Indication for lumbar puncture; 2) Cytological findings in subarachnoid hemorrhage; 3) Cytological findings in inflammatory CNS diseases; 4) Diagnostic interpretation; 5) Procedural knowledge; 6) and 7) Microscopic diagnosis of two case-based items. The seven pre-test questions are provided in the Supplementary Material 1.
2. Theoretical session (45 min): The lecture covered indications and contraindications for lumbar puncture, procedural principles, interpretation of laboratory parameters, characteristic cytological findings, and diagnostic aspects of common neurological diseases associated with pathological CSF findings.
3. Practical microscopy session (30 min): Students participated in the practical session in groups of six under the supervision of one instructor. Each student independently examined six standardized authentic CSF cytology preparations obtained from real patients using a light microscope. Students were first asked to identify and describe the observed cytological findings before interpreting their observations and assigning the most likely diagnosis. Following the individual assessment, the instructor facilitated a structured group discussion during which students compared their observations, explained their diagnostic reasoning, discussed the characteristic cytological features, and related the findings to common neurological diseases. The instructor subsequently summarized the key diagnostic features and provided individualized feedback. The session was designed as an active and experiential learning activity because students independently completed authentic diagnostic tasks rather than passively observing demonstrations. Procedural lumbar puncture skills training, simulation, and gamification were not included.
4. Post-test (20 min): Immediately following completion of the practical microscopy session, students completed a second paper-based pseudonymized multiple-choice test assessing the same knowledge domains using different questions. The seven post-test questions are also provided in the Supplementary Material 1.

### Knowledge assessment

The educational assessment consisted of two paper-based questionnaires: a pre-test administered before the intervention and a post-test administered immediately afterward. Each questionnaire contained seven multiple-choice questions covering the same seven predefined knowledge domains. Different but comparable questions were used in the pre- and post-tests to assess the same learning objectives while minimizing recall effects. Two questions evaluated recognition-level knowledge. Five application-level questions required students to interpret cytological findings, integrate microscopic observations with laboratory knowledge, or identify the most likely diagnosis based on authentic CSF preparations.

The questions were developed independently by the two neurologists who designed and delivered the course, both of whom have extensive experience in routine CSF diagnostics and undergraduate neurology teaching. The pre- and post-test questions were constructed to assess the same predefined learning objectives and cognitive levels while using different clinical scenarios and wording to minimize recall bias. The questions were reviewed jointly by both instructors before implementation to ensure comparable content coverage and difficulty. Because the instrument was developed for educational evaluation within a single course, formal psychometric validation or pilot testing was not performed.

Each correctly answered question was awarded one point (maximum score: 7 points). Total test scores were calculated for each participant. The percentage of participants among all enrolled students achieving each possible score was determined for both the pre-test and post-test. The primary outcome was the difference in the distribution of participant percentages across test scores between the pre-test and post-test. Secondary outcomes included performance within individual knowledge domains and student evaluations of the course.

### Student evaluation

Immediately after the session, students completed an anonymous course evaluation questionnaire. Participation in the evaluation survey was voluntary and had no influence on students’ course grades or academic evaluation. The evaluation consisted of four items assessing clarity of instruction, opportunities for participation, quality of feedback, and integration of theory and practice. Responses were recorded using a 5-point Likert scale ranging from strong disagreement (1) to strong agreement (5). An optional free-text field allowed additional comments. Evaluation results were analyzed descriptively. Reported percentages represent the proportion of students selecting the highest response category (5/5) relative to the total number of participating students. The English translation of the questionnaire is provided as Supplementary Material 2.

### Statistical analysis

Statistical analyses were performed using SPSS software for Windows (version 27.0; IBM Corp., Armonk, NY, USA). Pre- and post-test scores were analyzed as paired data because the same students completed both assessments. Score distributions were presented descriptively as the percentage of participants achieving each possible score. Since score distributions were non-normally distributed and ordinal in nature, differences between pre-test and post-test scores were analyzed using the Wilcoxon signed-rank test. Effect size was calculated as r = *Z*/**√***N*, where *Z* represents the standardized Wilcoxon test statistic and *N* the number of paired observations. For analyses of individual knowledge domains, each domain was represented by a single dichotomous outcome (correct vs. incorrect). Although different questions were used in the pre-test and post-test, each question pair was specifically designed to assess the same knowledge domain. Therefore, paired categorical outcomes were compared using McNemar’s test. A two-sided *p*-value < 0.05 was considered statistically significant.

## Results

### Overall performance following the educational intervention

A total of 84 medical students participated in the study, and all participants completed both the pre-test and post-test assessments.

**Fig. 1:**
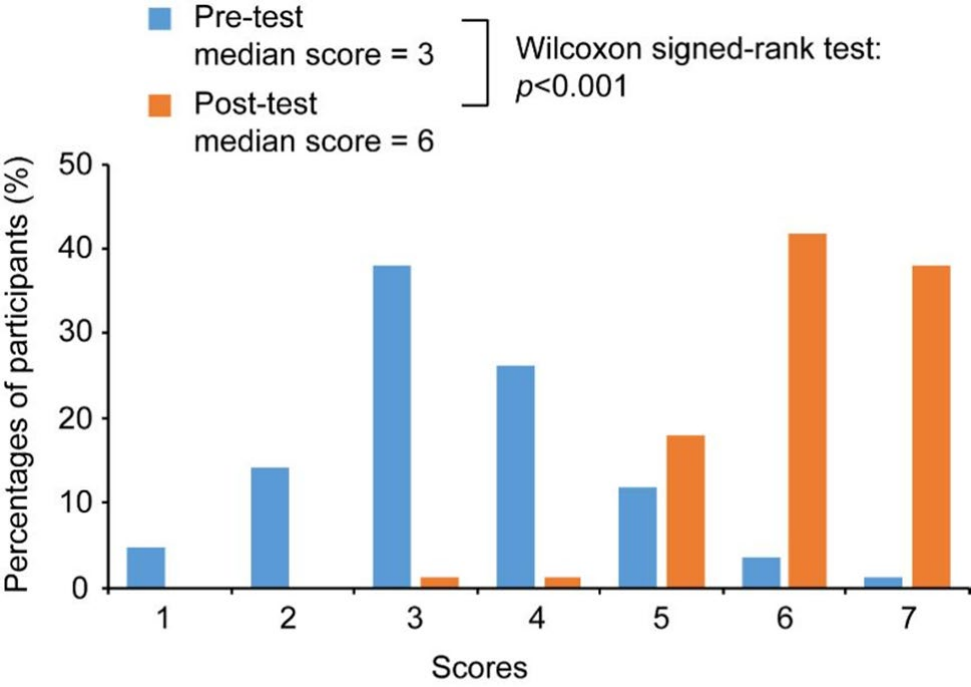
Improvement in overall test performance following the CSF diagnostics training module. The pre-test and post-test each consisted of seven questions, with one point awarded for each correctly answered question. The distribution of pre-test and post-test scores among all participating medical students is shown. Bars represent the percentage of students achieving each possible score. Error bars were not included because the data are presented as score distributions rather than continuous summary statistics. The median score increased from 3 before the practical course to 6 after the intervention. Paired analysis using the Wilcoxon signed-rank test demonstrated a statistically significant improvement in overall test performance following the training module (Z = − 7.886, *p* < 0.001; *n* = 84)

Overall performance improved following the educational intervention. The median test scores increased from 3 in the pre-test to 6 in the post-test (Fig. 1). In the pre-test, only 1.2% of participants (*n* = 1) answered all seven questions correctly, whereas this proportion increased markedly to 38.1% (*n* = 32) in the post-test. Likewise, the proportion of participants achieving six correct answers increased from 3.6% (*n* = 3) before the intervention to 41.7% (*n* = 35) after the intervention. Conversely, lower test scores became markedly less frequent following the intervention. The distribution of scores was shifted toward higher values in the post-test compared with the pre-test.

The paired comparison of pre- and post-test scores across all participants demonstrated that the practical course was associated with a significant improvement in overall performance (Wilcoxon signed-rank test for paired samples: Z = − 7.886, *p* < 0.001; Fig. 1). The intervention showed a large effect size (*r* = 0.86), according to conventional thresholds defining r values of 0.1, 0.3, and 0.5 as small, medium, and large effects, respectively.

### Domain-specific performance

We next evaluated performance across predefined knowledge domains represented by individual assessment items.

Competence in CSF diagnostics requires not only knowledge of indications and laboratory parameters, but also the ability to perform lumbar puncture appropriately and interpret cytological findings accurately. The educational intervention was associated with significant improvements across multiple knowledge domains, especially diagnostic reasoning, procedural knowledge, microscopic diagnosis and cytological interpretation.

Substantial gains were observed in procedural knowledge (question 5), with correct responses increasing from 18 in the pre-test to 74 in the post-test among all 84 participants (Table 1, *p* < 0.001). Similarly, the ability to interpret diagnostic constellations in neurological diseases (questions 2–4) improved significantly following the intervention (Table 1, all *p* values < 0.001). In addition, the proportion of students correctly identifying CSF cytology and establishing the correct diagnosis based on independent microscopic evaluation (questions 6 and 7) increased significantly (Table 1, *p* < 0.001 and *p* = 0.002 respectively).

**Table 1:** Improvement in performance on individual test items

Question	Domain	Cognitive category	Pre-test correct answer *n* (%)	Post-test correct answer *n* (%)	McNemar *p*-value
1	Indication for lumbar puncture	Recognition	71 (84.5%)	74 (88.1%)	0.629
2	CSF cytology (subarachnoid hemorrhage)	Application	22 (26.2%)	44 (52.4%)	< 0.001
3	CSF cytology (inflammatory disease)	Application	25 (29.8%)	80 (95.2%)	< 0.001
4	Diagnostic interpretation	Application	17 (20.2%)	81 (96.4%)	< 0.001
5	Knowledge of lumbar puncture procedures	Recognition	18 (21.4%)	74 (88.1%)	< 0.001
6	Microscopic diagnosis (subarachnoid hemorrhage)	Application	63 (75.0%)	80 (95.2%)	< 0.001
7	Microscopic diagnosis (bacterial meningitis)	Application	71 (84.5%)	83 (98.8%)	0.002

Pre-test and post-test questions differed but were designed to assess comparable content domains and cognitive demands. Values are presented as number of participants with correct answers (percentage). *p*-values were calculated using McNemar’s test for paired categorical data

Performance regarding indications for lumbar puncture showed no statistically significant change between assessments. Baseline performance for this domain was already high, which may have limited the ability to detect further improvement (71 correct responses in the pre-test vs. 74 in the post-test, *p* = 0.629).

### Student evaluation of the competency-based educational intervention

All 84 participants voluntarily completed the anonymous evaluation immediately following participation in the course. Evaluation results were presented as the proportion of participants selecting the highest response category (5/5) on the 5-point Likert scale. Overall, 71% of students reported that complex content was explained clearly, 70% indicated that active participation was encouraged, 76% agreed that they received helpful feedback, and 73% positively rated the integration of theory and practice (Table 2).

**Table 2:** Student evaluation of the CSF laboratory practical course

Item	Agreement (%)
Complex content explained clearly	71%
Active participation encouraged	70%
Helpful feedback provided	76%
Theory and practice well integrated	73%

Percentages represent the proportion of students selecting the highest response category (5/5) on the evaluation questionnaire

Optional free-text comments were reviewed descriptively. Suggestions for future development included the integration of lumbar puncture simulation training and the inclusion of additional case-based microscopy exercises. Based on this feedback, a lumbar puncture simulator was subsequently acquired for future teaching sessions.

## Discussion

Traditional medical education has historically emphasized the acquisition of theoretical knowledge, often focusing on the amount of information learned, course participation, and examination performance. In contrast, competency-based education prioritizes the development of practical and clinically applicable skills required for effective patient care [1, 9]. Within the field of neurology, conventional CSF teaching may primarily concentrate on memorizing indications for lumbar puncture or recalling characteristic CSF parameters. However, clinical competence in CSF diagnostics requires substantially more than factual knowledge alone. It requires integration of cytological findings with the clinical context. Undergraduate neurology education should develop both procedural and analytical competencies, not procedural skills alone [7]. Our study has shown that medical students are able to learn how to recognize pathological cytological findings, interpret diagnostic results, and apply this knowledge to clinically relevant diagnostic scenarios [10].

This study evaluated the educational impact of a structured, hands-on CSF diagnostics training module integrated into the undergraduate neurology curriculum. Participation in the intervention was associated with improved short-term knowledge acquisition and application of diagnostic knowledge, particularly in domains requiring interpretation of CSF findings, cytological assessment, procedural knowledge, and microscopy-based diagnosis.

The observed improvement may be explained by the combination of theoretical instruction and active engagement with authentic diagnostic material. Educational research has consistently shown that active learning approaches can enhance learner engagement and support application of knowledge compared with exclusively lecture-based formats [2, 3]. In our intervention, students were not limited to passive observation but were required to independently describe cytological findings, interpret microscopic observations, and assign a likely diagnosis before participating in instructor-guided discussion. This structure aligns with experiential learning concepts that emphasize active participation, reflection, and application of knowledge within authentic learning contexts [11–13]. Similar effects have been reported in simulation-based and skills-oriented training, where practice-based learning environments improve both diagnostic accuracy and learner confidence [5, 14]. Furthermore, the use of pre- and post-testing may have contributed to the observed learning gains through test-enhanced learning mechanisms, which are known to strengthen knowledge retention and retrieval [15].

The largest improvements were observed in domains requiring interpretation and integration of findings rather than factual recall alone. This observation may suggest that exposure to authentic microscopy material and structured discussion supported students in connecting theoretical concepts with practical diagnostic tasks. Importantly, the educational outcomes reported in this study reflect changes in short-term knowledge acquisition and application of diagnostic knowledge rather than direct assessment of clinical competence or procedural performance.

Performance regarding indications for lumbar puncture did not improve significantly following the intervention. One possible explanation is a ceiling effect, as baseline performance in this domain was already high prior to participation. This finding may reflect concurrent exposure to neurology lectures during the semester and suggests that the greatest educational value of the intervention may lie in domains that are less frequently addressed through traditional lecture-based instruction.

Student evaluation findings indicated favorable perceptions of the educational format, particularly regarding integration of theoretical and practical learning elements. Optional free-text comments additionally identified opportunities for further curriculum development. Students suggested inclusion of lumbar puncture simulation and expansion of case-based microscopy exercises. In response to this feedback, a lumbar puncture simulator was subsequently incorporated into the local teaching program for future iterations of the course [5].

This study adds to the existing literature by focusing specifically on CSF diagnostics, an area that is highly relevant in clinical practice but for which published reports describing structured undergraduate teaching approaches remain limited. Previous undergraduate educational studies have predominantly focused on improving students’ confidence and technical performance in lumbar puncture through simulation-based teaching [5, 6]. In contrast, our intervention addressed a different educational objective by focusing on interpretation of authentic CSF findings and diagnostic reasoning. Rather than replacing procedural simulation, microscopy-based diagnostic training complements procedural skills training by developing students’ ability to interpret authentic CSF findings and integrate them into diagnostic reasoning. The present intervention combines theoretical instruction with direct, hands-on analysis of real CSF specimens, thereby closely reflecting clinical diagnostic workflows. The pronounced improvements observed in domains such as cytological interpretation and diagnostic reasoning suggest that this approach may be particularly useful for supporting learning of complex, pattern-based diagnostic tasks. Furthermore, the high level of student acceptance indicates that such practice-oriented formats are not only effective but also feasible for integration into existing curricula. These findings support the incorporation of structured, hands-on diagnostic training modules as a complement to traditional teaching methods in neurology education. These findings are consistent with current recommendations in undergraduate neurology education, which emphasize the development of analytical skills and diagnostic reasoning alongside procedural training, and support recent calls for more authentic, competency-oriented educational interventions in neurology curricula [1, 4].

Several limitations should be considered when interpreting these findings. First, the study did not include a control group, limiting the ability to attribute improvements solely to the intervention. Second, educational outcomes were measured using short-term written assessments immediately following the intervention and therefore do not permit conclusions regarding long-term retention or transfer into clinical performance. Third, the intervention was conducted at a single institution within one curricular setting, which may limit generalizability. Fourth, because practical procedural skills were not directly assessed, conclusions should be restricted to knowledge acquisition and application of diagnostic knowledge rather than procedural competence. Fifth, improvements were measured using written assessments rather than objective structured clinical examinations or real-world clinical performance measures. In addition, the assessment instruments were developed locally and were not formally validated.

Despite these limitations, the study demonstrates that structured integration of theoretical instruction with authentic microscopy-based learning can be implemented within undergraduate neurology education and may support clinically relevant learning experiences. Future studies should investigate long-term retention, transfer to clinical performance, and integration of procedural simulation into CSF teaching formats.

## Conclusions

A structured, hands-on CSF diagnostics training module integrated into undergraduate neurology education was associated with improved short-term knowledge acquisition and application of diagnostic knowledge among medical students. The combination of theoretical instruction with microscopy-based examination of authentic CSF preparations provided an opportunity for students to actively interpret findings and apply diagnostic concepts within a clinically relevant context. These findings suggest that practice-oriented educational approaches may complement traditional lecture-based teaching in neurology education. Further research is needed to evaluate long-term retention of learning outcomes, transfer into clinical performance, and the educational impact of integrating procedural simulation into CSF teaching.

## Supplementary Information

Supplementary Material 1.

Supplementary Material 2.

## Data Availability

All data generated or analyzed during this study are included in this published article and available from the corresponding author on reasonable request.

## Abbreviations

CSF: Cerebrospinal fluid
