# Supplementary material for: A structured hands-on CSF diagnostic training module improves diagnostic knowledge in medical students: a prospective pre–post study
Source: BMC Med Educ. 2026 Jul 22;26:1197. doi: 10.1186/s12909-026-09990-2 (PMC13393871; doi:10.1186/s12909-026-09990-2)
Supplement: Supplementary file 1 — Supplementary Material 1. [file 12909_2026_9990_MOESM1_ESM.docx]

**Student Course Evaluation – CSF Diagnostics Practical Course**

Instruction:
Please indicate your level of agreement with the following statements.

Response options:
1 = Strongly disagree
2 = Disagree
3 = Neutral
4 = Agree
5 = Strongly agree

The course content was presented in a clear and understandable manner.

| Strongly disagree | 1 | 2 | 3 | 4 | 5 | Strongly agree |
| --- | --- | --- | --- | --- | --- | --- |

The course encouraged active participation.

| Strongly disagree | 1 | 2 | 3 | 4 | 5 | Strongly agree |
| --- | --- | --- | --- | --- | --- | --- |

The instructor provided constructive and helpful feedback.

| Strongly disagree | 1 | 2 | 3 | 4 | 5 | Strongly agree |
| --- | --- | --- | --- | --- | --- | --- |

The combination of theoretical instruction and practical microscopy supported my learning.

| Strongly disagree | 1 | 2 | 3 | 4 | 5 | Strongly agree |
| --- | --- | --- | --- | --- | --- | --- |

Optional free-text comment:
Please provide any additional comments or suggestions for improvement.

|  |
| --- |
