# Supplementary material for: A structured hands-on CSF diagnostic training module improves diagnostic knowledge in medical students: a prospective pre–post study
Source: BMC Med Educ. 2026 Jul 22;26:1197. doi: 10.1186/s12909-026-09990-2 (PMC13393871; doi:10.1186/s12909-026-09990-2)
Supplement: Supplementary file 2 — Supplementary Material 2. [file 12909_2026_9990_MOESM2_ESM.docx]

**Test 1 (Pre-test)**

**Question 1**

Which answer is incorrect?
Typical indications for cerebrospinal fluid (CSF) diagnostics include the detection or exclusion of the following conditions:

A. Meningoencephalitis
B. Neoplastic meningiosis
C. Subarachnoid hemorrhage
D. Neurodegenerative dementia
E. Ischemic stroke

**Question 2**

Which answer is incorrect?
Diagnostic findings relevant in or after subarachnoid hemorrhage include the detection of:

A. Erythrocytes
B. Erythrophages
C. Free hemosiderin and bilirubin crystals
D. Thrombocytophages
E. Leukophages

**Question 3**

Which answer is incorrect?
Typical findings in inflammatory diseases of the central nervous system include the detection of:

A. Band and segmented neutrophils in acute bacterial meningitis
B. Segmented neutrophils in acute viral meningoencephalitis
C. Predominantly lymphocytes in subacute viral meningoencephalitis
D. Plasma cells in neuroborreliosis
E. Neutrophilic granulocytes in multiple sclerosis

**Question 4**

Which answer is incorrect?
A CSF cell count of 240 cells/µL is typically compatible with the following diagnoses:

A. Neoplastic meningiosis
B. Multiple sclerosis
C. Acute neuroborreliosis
D. Herpes simplex encephalitis
E. Tuberculous meningoencephalitis

**Question 5**

Which answer is incorrect?
Regarding lumbar puncture:

A. The appropriate intervertebral space is at the L4/L5 level
B. An atraumatic needle should preferably be used
C. Measurement of CSF opening pressure is possible both in the supine and sitting position
D. The procedure is contraindicated when Quick <50%
E. Simultaneous blood sampling is necessary

**Question 6**


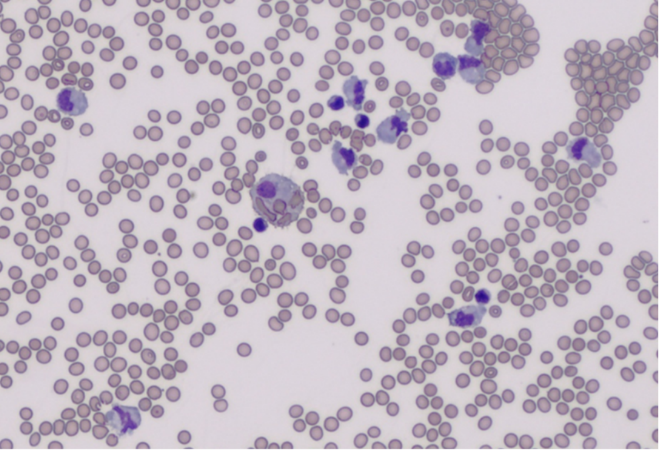


Which diagnosis is most typically associated with this cytological image?

A. Meningitis
B. Neoplastic meningiosis
C. Subarachnoid hemorrhage
D. Alzheimer’s dementia

E. Multiple Sclerosis

**Question 7**


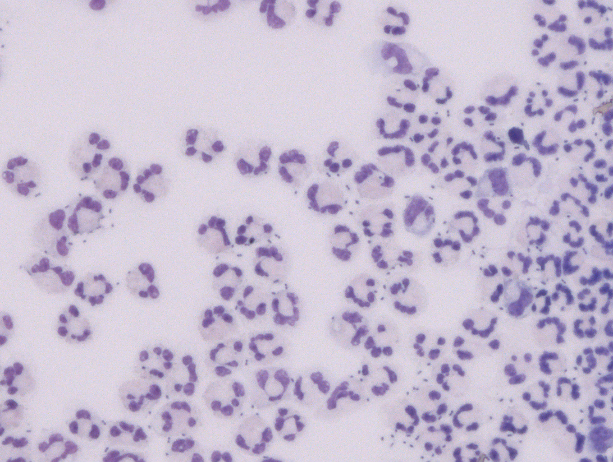


Which diagnosis is most typically associated with this cytological image?

A. Meningitis
B. Neoplastic meningiosis
C. Subarachnoid hemorrhage
D. Alzheimer’s dementia
E. Ischemic stroke

**Answers: EDEBCCA**

**Test 2 (Post-test)**

**Question 1**

Which answer is incorrect?
Typical indications for cerebrospinal fluid (CSF) diagnostics include the detection or exclusion of the following conditions:

A. Meningoencephalitis
B. Neoplastic meningiosis
C. Subarachnoid hemorrhage
D. Idiopathic intracranial hypertension
E. Intracranial hemorrhage in the typical location

**Question 2**

Which answer is incorrect?
Diagnostic findings relevant in or after subarachnoid hemorrhage include the detection of:

A. Erythrocytes
B. Erythrophages
C. Free hemosiderin and bilirubin crystals
D. Granulocytes
E. Leukophages

**Question 3**

Which answer is incorrect?
Typical findings in inflammatory diseases of the central nervous system include the detection of:

A. Predominantly segmented neutrophils in acute bacterial meningitis
B. Diplococci in pneumococcal meningitis
C. Activated lymphocytes and monocytes in viral meningoencephalitis
D. Plasma cells in neuroborreliosis
E. Activated lymphocytes in Alzheimer’s dementia

**Question 4**

Which answer is correct?
A CSF cell count of 2000 cells/µL is typically compatible with the following diagnoses:

A. Neoplastic meningiosis
B. Multiple sclerosis
C. Varicella-zoster virus encephalitis
D. Herpes simplex encephalitis
E. Bacterial meningoencephalitis

**Question 5**

Which answer is incorrect?
Regarding lumbar puncture:

A. The appropriate intervertebral space is at the L4/L5 level
B. An atraumatic needle should preferably be used
C. The procedure is possible with Quick >50%, PTT <50 s, and platelet count >50,000/µL
D. The procedure is possible in the presence of signs of elevated intracranial pressure without prior imaging
E. Simultaneous blood sampling is necessary

**Independent microscopy assessment**

**Question 6**

**A standardized authentic CSF cytology preparation obtained from a real patient is provided.**

Examine the CSF cytology preparation. Based on the microscopic findings, which of the following is the **most likely diagnosis**?

A. Meningoencephalitis
B. Neoplastic meningiosis
C. Subarachnoid hemorrhage
D. Alzheimer’s dementia
E. Ischemic stroke

**Question 7**

**A standardized authentic CSF cytology preparation obtained from a real patient is provided.**

Examine the CSF cytology preparation. Based on the microscopic findings, which of the following is the **most likely diagnosis**?

A. Meningoencephalitis
B. Neoplastic meningiosis
C. Subarachnoid hemorrhage
D. Alzheimer’s dementia
E. Ischemic stroke

**Answers: EDEEDCA**
